# Sudden Cardiac Death in a Case of Non-Dominant Coronary Artery Obstruction Without Depressed Left Ventricular Function

**DOI:** 10.4021/cr272e

**Published:** 2013-07-11

**Authors:** Hung Yi Chen

**Affiliations:** Department of Cardiology, Taipei City Hospital-Heping Branch, No. 33, Sec. 2, Zhonghua Rd., Taipei City 100, Taiwan. Email: anigi426@ms24.hinet.net

**Keywords:** Sudden cardiac death, Left circumflex coronary artery, Acute myocardial infarction, Atrial fibrillation

## Abstract

Acute myocardial infarction complicated with lethal cardiac arrhythmia remains the major cause of sudden death. The possible clinical presentation leading to lethal ventricular arrhythmia has been demonstrated but the data are limited. The previous study revealed no significant correlation between sudden cardiac death and the location of coronary obstruction site. And the possible mechanism of sudden cardiac death in non-dominant coronary artery obstruction is unclear. We presented a case of acute myocardial infarction with mid left circumflex artery occlusion complicated with new onset atrial fibrillation initially. The rhythm degenerated into ventricular fibrillation immediately and sudden cardiac death occurred. After resuscitation, he received coronary angioplasty, and the rhythm recovered to sinus after the occluded coronary artery reopened. We thick new onset atrial fibrillation could be a potential risk factor leading to sudden death in acute myocardial infarction with obstruction of non-dominant coronary artery. Control of ventricular rate and early restoration of sinus rhythm may be potential benefit.

## Introduction

Sudden death is common in acute myocardial infarction (MI). Acute myocardial ischemic injury resulting in lethal arrhythmia is the major mechanism. The more extend of coronary artery disease may result in larger amount of myocardial injury and share the higher VF occurrence. However, sudden cardiac death in non-dominant coronary artery obstruction was found by autopsy. And the previous study revealed no significant correlation between sudden cardiac death and the location of coronary obstruction site. We reported a case of acute myocardial infarction complicated with new onset atrial fibrillation initially, and the rapid ventricular rhythm induced ventricular fibrillation. After reopening the left circumflex artery (infarct related artery), his rhythm converted to sinus again. We think new onset atrial fibrillation could be a potential lethal arrhythmia in acute myocardial infarction, even with obstruction of non-dominant coronary artery. We described the case and reviewed the articles.

## Case Report

**Figure 1:**
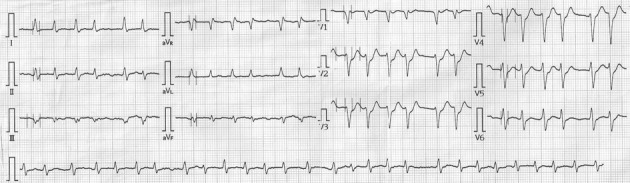
The 12-lead electrocardiogram at emergency department shows atrial fibrillation with ST segments depression in precordial leads.

**Figure 2:**
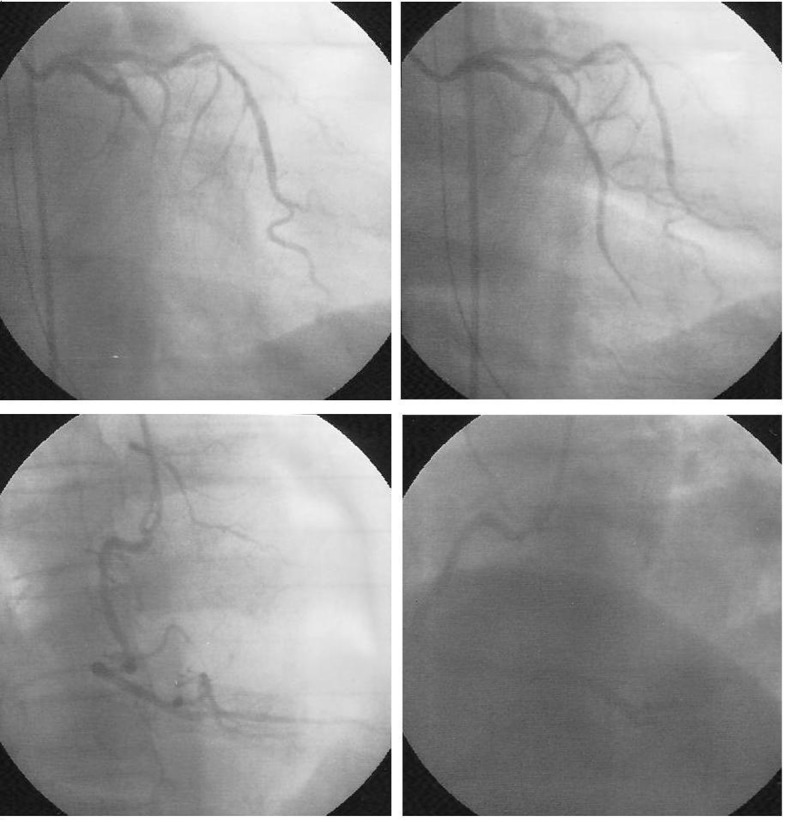
(Upper panel) coronary angiography of left coronary artery shows total occlusion on the mid portion of left circumflex artery (left), and after stent deployed (right); (lower panel) coronary angiography of right coronary artery.

A 62-year-old man was admitted to our emergency department (ED) complaining of chest pain on exertion for near two weeks and symptom had deteriorated for two days. He had a history of systemic hypertension and diabetes as risk factors for coronary artery disease without regular medication controlled. He had no history of tobacco or alcohol use. His blood pressure was 152/82 mmHg, and pulse rate was 77 beats/min on arrival. Physical examination of the cardiovascular system was unremarkable. His resting electrocardiogram (ECG) showed sinus rhythm without obvious ST segment and T wave changes. Biochemical analysis demonstrated slightly elevation levels of troponin I (0.17 µg/L, reference range, 0 - 0.04 µg/L), total creatine kinase (CK, 160 U/L; reference range, 30 - 170 U/L) and CK-myocardial isoenzyme (CK-MB, 19 U/L; reference range, 0 - 16 U/L). Admission was suggested under the suspicion of acute coronary syndrome. Six hours later, follow-up laboratory data showed deterioration of troponin I: 0.23 µg/L, CK 275 U/L, and CK-MB 36 U/L. Echocardiography demonstrated good left ventricular contractility. Neither significant regurgitation nor pericardial effusion was detected. Treatment with antihypertension agents, anticoagulants, and nitrate was initiated, and catheterization was arranged on the following day. The results showed atherosclerotic change of left coronary artery including left anterior descending artery (LAD) and left circumflex artery (LCX) with a critical lesion (more than 90% obstruction) on mid portion of LCX. No significant obstruction found in right coronary artery (RCA). Then angioplasty for mid LCX lesion was performed. After several times of ballooning, the result was suboptimal. We did not deploy stent initially because of the relative small caliber of coronary artery. The patient was well and kept symptom free. He was discharged from our hospital on the fifth days after admission. He did not take follow-up after discharge. Thirteen days later, he was sent to our ED again under complaining of chest pain with cold sweating. His blood pressure was 186/96 mmHg. His ECG demonstrated atrial fibrillation (AF) rhythm with ST segment depression in precordial leads (Fig. 1). Initial biochemical analysis at ED demonstrated normal CK level (134 U/L), and border levels of troponin I (0.04 µg/L), and CK-MB (21 U/L). Other laboratory data as renal function test and electrolyte were non-remarkable (sodium: 136 mEq/L, potassium: 3.9/mEq/L, calcium: 9.2 mg/dL). Acute coronary syndrome was impressed, and he was treated with aspirin, clopidogrel and anticoagulant. Twenty minutes later, the AF degenerated into ventricular fibrillation (VF). Hemodynamic collapsed suddenly and the patient lost his consciousness. Resuscitation with defibrillation shock and intubation were given immediately. After his rhythm returned to AF, the patient was sent to our intensive care unit for further care. He was treated with amiodarone infusion to avoid malignant arrhythmia. Cardiac enzyme was re-checked four hours later at our intensive care unit, and it showed elevation of troponin I (2.35 0.04 µg/L), CK (1170 U/L) and CK-MB (186 U/L) levels. Under the suspicion of acute myocardial infarction, the patient received coronary angiography and the results showed LCX was totally occlusion on its mid portion where was the site of previous intervention. No further obstruction was found in LAD and RCA. Repeat angioplasty with a 2.5 × 23 stent deployed on the lesion site was performed (Fig. 2). His rhythm recovered to sinus rhythm after the procedure. Repeat echocardiography showed fair left ventricular function and the patient was discharged two days later with excellent condition.

## Discussion

Sudden death is common in acute myocardial infarction (MI). VF is known to be the major complication after acute MI. The occurrence of lethal arrhythmia in acute MI results from acute ischemic injury producing electrical dysfunction. It is well known that old age, large infarction size and higher killip class are frequently associated with the lethal ventricular arrhythmia after hospitalized with acute MI [1]. The more extend of coronary artery disease may result in larger amount of myocardial injury and share the higher VF occurrence. This is evidenced that patients with early primary VF were more likely to have initial electrocardiographic evidence of large to extensive ischemic injury, and the size of the ischemic area is related to the incidence of early VF [2]. The size of ischemic area is not the absolute potential risk factors associated with ventricular arrhythmia. Other factors as young age, female gender, absence of diabetes history and inferior infarct location had been reported to be potential risk factors for primary ventricular fibrillation in acute MI [3, 4]. The leading factors of lethal arrhythmia in acute MI are inconsistent in different studies. This is because the electrophysiological mechanism of VF is the result from complex interactions between myocardial injury, autonomic tone and ionic status of myocardium [5, 6]. In addition, more than half of deaths associated with acute MI developed within the first hour, usually before any detective monitor can be available. Therefore, the complete survey may be limited. Until now, the potential risk factors and the mechanisms of sudden death during the onset of acute myocardial ischemia remain unclear.

Associations between primary ventricular fibrillation and the site of myocardial infarction had been studied and the results showed conflict. As to the relationship between the culprit coronary artery and sudden cardiac death, there have been very rare studies. Gheeraert et al tried to understand whether there were relationships between the location of coronary obstruction site and out-of-hospital ventricular fibrillation in acute myocardial infarction. In comparing 72 patients complicated by out-of-hospital VF with 144 without VF within one hour after symptom onset, the results showed occlusion in the left coronary artery (LCA) was associated with greater risk for out-of-hospital VF than the RCA in acute MI. In their study, the results also showed non-significant correlation between out-of-hospital VF with the location of occlusion within LCA, amount of myocardial necrosis and extend of coronary artery disease [7]. Recently, Geske et al tried to demonstrate the precise location of culprit lesions in acute MI at autopsy in 41 non-diabetic patients. They found most of the culprit lesions in LCA occurred within proximal 3.0 cm of the LAD and the LCX. In contrast, culprit plaques in the RCA were distributed evenly throughout it length [8]. Again, because of difficulty in sampling, the results remain inconsistent.

AF is a relatively common complication in acute MI with various incidences of 6-21% of hospitalized patients [9]. The early new onset AF rhythm may occur alone or in associated with other complications, and generally thought to be a marker of adverse prognosis. Not only at a greater risk for acute stroke, new onset AF also had higher rates of all cause in-hospital complications as reinfarction, shock, heart failure and ventricular arrhythmia [10, 11]. And they shared a more than threefold increased risk for death in patients with acute coronary syndrome compared with those who did not develop AF [11, 12]. The occurrence of AF rhythm can be caused by atrial ischemia, atrial distension secondary to the left ventricular failure or significant diastolic ventricular dysfunction [13]. The possible predisposing factors of AF after acute MI as advanced age, severe left ventricular dysfunction, valvular impairment had been reported [10]. In spite of autonomic tone and endogenous catecholamines may also precipitate AF, most of the patients with new onset AF in acute coronary syndrome were in associated with other co-morbidity conditions. Therefore, new onset AF may simply reflect myocardial disease significant enough to cause arrhythmia. This indicates the development of AF can serve as a marker for greater myocardial dysfunction rather than an independent predictable factor of poor outcomes in acute MI.

Whether there is relationship between location of culprit coronary artery and AF occurrence in acute MI is currently controversial. In analysis of GUSTO-III study, the results showed no significant difference between new onset of the AF and location of MI (anterior or inferior) in 906 patients [11]. Some studies demonstrated new onset AF was more prevalent when culprit coronary artery was LAD than RCA or LCX [14]. Different from above, another study showed LCX involvement was more common in patients with AF in acute MI [15]. Furthermore, a previous study had been documented that early AF might develop in acute MI when obstruction of the proximal left circumflex artery and implied this could result in left atrial ischemia and impairment of the perfusion to the atrioventricular nodal artery [13]. This might be an explanation of the occurrence of AF in LCX lesion as our case, although we did not obtain the electrocardiographic evidence of Af development, Different from our case, since the atrioventricular nodal artery originates from RCA in 90% and from LCX in only 10% of normal subjects, the authors claimed that early AF will most often require coexistent total or near total occlusion of both right and the proximal left circumflex coronary arteries. Therefore, the new onset of AF after acute MI generally indicated at least two-vessel coronary artery disease. However, if AF in acute MI was mainly induced by atrial ischemia, reopen the critical coronary artery may resolve the problem. Similar to our case, AF was converted into sinus rhythm by angioplasty in LCX in a patient with acute MI demonstrated by Bunc et al [16].

The occurrence of AF in acute MI might exacerbate myocardial ischemia. This is particular important because rapid irregular ventricular stimulation may deteriorate left ventricular function and impair coronary perfusion and result in severe ventricular arrhythmia developing in vulnerable ischemic myocardium [17]. We tried to emphasize the occurrence of AF in the setting of acute MI might present a warning event requiring immediate intervention. And it could be the potential risk factor leading to cardiac death even in non-dominant coronary obstruction.

In summary, we do not know the true possibility and precise data between new onset AF and sudden cardiac death. Our case suggests AF is not only in the setting of acute MI as a high risk finding, it also represents a potentially very treatable risk, even transient. The management should include intensive surveillance, mediation controlled, and cardioversion whenever possible. 
